# Joint association of TyG index and high sensitivity C-reactive protein with cardiovascular disease: a national cohort study

**DOI:** 10.1186/s12933-024-02244-9

**Published:** 2024-05-07

**Authors:** Cancan Cui, Lin Liu, Yitian Qi, Ning Han, Haikun Xu, Zhijia Wang, Xinyun Shang, Tianjiao Han, Yining Zha, Xin Wei, Zhiyuan Wu

**Affiliations:** 1 Department of Radiology, China-Japan Union Hospital of Jilin University, Jilin University, Changchun, China; 2 Harvard T H Chan School of Public Health, Boston, USA; 3 Centre for Precision Health, School of Medical and Health Sciences, Edith Cowan University, 270 Joondalup Drive, Joondalup, WA 6027 Australia

**Keywords:** Triglyceride-glucose (TyG) index, Insulin resistance, Inflammation, High-sensitivity C-reactive protein (hsCRP), Cardiovascular disease

## Abstract

**Background:**

Both the triglyceride-glucose (TyG) index, as a surrogate marker of insulin resistance, and systemic inflammation are predictors of cardiovascular diseases; however, little is known about the coexposures and relative contributions of TyG index and inflammation to cardiovascular diseases. Using the nationally representative data from the China Health and Retirement Longitudinal Study (CHARLS), we conducted longitudinal analyses to evaluate the joint and mutual associations of the TyG index and high-sensitivity C-reactive protein (hsCRP) with cardiovascular events in middle-aged and older Chinese population.

**Methods:**

This study comprised 8 658 participants aged at least 45 years from the CHARLS 2011 who are free of cardiovascular diseases at baseline. The TyG index was calculated as Ln [fasting triglyceride (mg/dL) × fasting glucose (mg/dL)/2]. Cardiovascular events were defined as the presence of physician-diagnosed heart disease and/or stroke followed until 2018.We performed adjusted Cox proportional hazards regression and mediation analyses.

**Results:**

The mean age of the participants was 58.6 ± 9.0 years, and 3988 (46.1%) were females. During a maximum follow-up of 7.0 years, 2606 (30.1%) people developed cardiovascular diseases, including 2012 (23.2%) cases of heart diseases and 848 (9.8%) cases of stroke. Compared with people with a lower TyG index (< 8.6 [median level]) and hsCRP < 1 mg/L, those concurrently with a higher TyG and hsCRP had the highest risk of overall cardiovascular disease (adjusted hazard ratio [aHR], 1.300; 95% CI 1.155–1.462), coronary heart disease (aHR, 1.294; 95% CI 1.130–1.481) and stroke (aHR, 1.333; 95% CI 1.093–1.628), which were predominant among those aged 70 years or below. High hsCRP significantly mediated 13.4% of the association between the TyG index and cardiovascular disease, while TyG simultaneously mediated 7.9% of the association between hsCRP and cardiovascular risk.

**Conclusions:**

The findings highlight the coexposure effects and mutual mediation between the TyG index and hsCRP on cardiovascular diseases. Joint assessments of the TyG index and hsCRP should be underlined for the residual risk stratification and primary prevention of cardiovascular diseases, especially for middle-aged adults.

**Supplementary Information:**

The online version contains supplementary material available at 10.1186/s12933-024-02244-9.

## Background

Cardiovascular disease (CVD) is the largest contributor to global mortality [1]. In China, the prevalence of CVD has doubled since 1990, reaching nearly 94 million in 2016 [2]. There is an urgent need to understand the current epidemiological features and risk factors for the major types of CVD and the implications of these features for the primary prevention of CVD. Conventional risk factors in the Framingham risk score (FRS), such as age, cholesterol, hypertension, and smoking, account for most of the risk of CVD and have been used for risk assessment for decades [3]. However, approximately one-third of individuals with fewer risk factors develop CVD, and approximately 40% of individuals with low cholesterol levels die from coronary arterial events in the general population [4–6], highlighting the residual CVD risk [7]. Therefore, a wide array of other biomarkers for the refinement of risk assessment of CVD are promising.

Insulin resistance, characterized by a decrease in sensitivity or responsiveness to the metabolic actions of insulin, including insulin-mediated glucose disposal, has been widely identified as an independent risk factor for the development of CVD. The attributable risk to diabetes is relatively low than 10% [8], and that there is conflicting data on whether accounting for insulin resistance could further improve CVD risk prediction among different populations [9]. There is an acceleration of the incidence and prevalence of insulin resistance along with associated CVD [10]. The triglyceride-glucose (TyG) index has been proposed as a reliable surrogate indicator of insulin resistance [11, 12], which also plays an important role in CVD [13]. Recently, a considerable number of studies have provided strong evidence suggesting the predictive effect of the TyG index on CVD [14–16]. In addition, the TyG index is also closely associated with risk factors for cardiovascular diseases, such as arterial stiffness and hypertension [17, 18]. Notably, multifactorial risk factor evaluation and management are essential for the early prevention of CVD [19]. Systemic inflammation is often observed in CVD incidence [20]. A large body of evidence has accumulated supporting the use of high-sensitivity C-reactive protein (hsCRP) as a clinical measure of inflammation, which holds promise to enable risk stratification of CVD in clinical practice [21]. Previous studies have highlighted the need for combined assessment and management of chronic inflammation and atherogenic dyslipidemia in the primary prevention of CVD [20, 22]. A recent study found that adding hsCRP and TyG index to conventional risk model improved the risk reclassification for CVD, while there is no significant interaction between hsCRP and TyG index [23]. Our previous study suggested the combined evaluation of the TyG index and renal function for the risk reclassification of CVD, in which impaired renal function partially mediated the association between the TyG index and cardiovascular risk [24]. However, the mutual association between insulin resistance, inflammation and CVD remains unclear, given the possible mutual mediating effect between insulin resistance and inflammation in terms of CVD [25, 26].

To fill this knowledge gap, we therefore conducted a longitudinal study based on data from a prospective national cohort to examine the joint effect and risk reclassification capacity of the TyG index and hsCRP level with the onset of CVD. Together, we conducted a mediation analysis to underline the mutual mediating effect linking the TyG index and hsCRP level to the development of CVD.

## Methods

### Data source and study population

This current study was a secondary analysis of the China Health and Retirement Longitudinal Study (CHARLS), which is a national population-based cohort study (http://charls.pku.edu.cn/) among Chinese adults aged 45 years or older initiated in 2011. The following health surveys were issued every 2 years, with a total of four surveys through 2018. The participants were recruited from both rural and urban areas using a multistage stratified probability proportional-to-size sampling strategy and covered 150 counties or districts of 28 provinces in China. Details of the study design and cohort profile have been previously described [27].

At each survey, sociodemographic characteristics, medical history, and lifestyle behaviors were collected by the trained staff by face-to-face interviews using a standardized questionnaire [28]. In this current study, participants who underwent the first visit (2011–2012) were included as baseline and then followed at the three subsequent surveys (2013–2014, 2015–2016, 2017–2018). Those lacking necessary sociodemographic characteristics (age and sex), blood sample tests or data of cardiovascular diseases or cancer history at baseline were excluded. Finally, a total of 8 658 participants were included in the final analysis. The detailed inclusion and exclusion process is shown in Additional file 1: Fig. S1.

The CHARLS study was performed in accordance with the principles of the Declaration of Helsinki and was approved by the Institutional Review Board of Peking University (IRB00001052-11015). All participants provided written informed consent before participating in the CHARLS study. This study was conducted following the Strengthening the Reporting of Observational Studies in Epidemiology (STROBE) reporting guidelines.

### Exposure

Fasting venous blood samples were collected by medical staff from the Chinese Centre for Disease Control and Prevention based on the standard protocol and subsequently tested at the central laboratory. Triglycerides and glucose were measured based on an enzymatic colorimetric test. The coefficient of variation of triglycerides was 1.5% within the assay. The coefficient of variation of glucose was 0.9% within the assay. The concentration of hsCRP was measured based on an immunoturbidimetric assay on a Hitachi 7180 chemistry analyzer (Hitachi, Tokyo, Japan). The coefficient of variation (CV) of blood marker measurement was < 5%. According to previous studies [16, 29], the TyG index was calculated as Ln [triglycerides (mg/dL) × glucose (mg/dL)/2].

### Ascertainment of CVD events

The study outcome was the incidence of CVD events. In accordance with previous studies [30, 31], incident CVD events were assessed by the following standardized questions: “Have you been told by a doctor that you have been diagnosed with a heart attack, coronary heart disease, angina, congestive heart failure, or other heart problems?” or “Have you been told by a doctor that you have been diagnosed with a stroke?” Participants who reported heart disease or stroke during the follow-up period were defined as having incident CVD. The date of CVD diagnosis was recorded as being between the date of the last interview and that of the interview reporting an incident CVD. The outcomes were assessed by rigorously trained interviewers through standardized questionnaires that are harmonized to international leading aging surveys in the Health and Retirement Study (HRS) and related international aging surveys, including the English Longitudinal Study of Aging (ELSA) and the Survey of Health, Aging and Retirement in Europe (SHARE). Quality control of data recording and checking was conducted to ensure data reliability.

### Covariates

Baseline measurements of age, sex, education level, marital status, residence location, BMI, smoking, drinking and self-reported health conditions (hypertension and diabetes) were included as covariates in the current study. Educational level was categorized as “primary education,” “secondary education,” and “third education.” Marital status included “married” and “others.” Residence location included “urban” and “rural.” Smoking status was defined as “never smoking”, “current smoker” and “former smoker”. BMI was calculated as weight (in kilograms)/height^^2^ (in meters squared) [32]. Hypertension was defined as systolic blood pressure ≥ 140 mmHg or diastolic blood pressure ≥ 90 mmHg or self-reported diagnosis history of hypertension or use of any antihypertensive medication [33]. Diabetes was defined as fasting glucose ≥ 7.0 mmol/L or self-reported diagnosis history of diabetes or use of any hypoglycemic medication [34].

### Statistical analysis

Data are described as the means and standard deviation (SD) for continuous variables. Frequency with percentage was used to describe categorical variables. Baseline characteristics are summarized according to joint assessments of the TyG index (median value [8.6] as cutoff point) and hsCRP (1 mg/L as cutoff point) and compared among participants of four groups (TyG < median & hsCRP < 1 mg/L, TyG < median & hsCRP ≥ 1 mg/L, TyG ≥ median & hsCRP < 1 mg/L and TyG ≥ median & hsCRP ≥ 1 mg/L) using the χ^2^ test or analysis of variance, as appropriate. Due to lacking clinical cutoff point of TyG, we used the median value of TyG following previous studies [23, 24], and TyG levels above median should be interpreted as elevated values. Eighteen percent (1532 of 8658) of total data items were missing and were assumed to be missing at random.

We computed the person-time of follow-up for each participant from the date of the 2011 to 2012 survey (baseline) to the dates of the CVD diagnosis or the end of follow-up (2017 to 2018 survey), whichever came first. Incidence rates of CVD events per 1000 person-years were calculated. To determine the association of the TyG index and hsCRP with the development of CVD, multivariable-adjusted Cox proportional hazards models were used to calculate the hazard ratio (HR) with 95% confidence interval (CI), considering the time-to-event framework. The proportional hazards assumption was tested using Schoenfeld residuals, and no potential violation was observed. Age (continuous) and sex were adjusted in model 1, and in model 2, residence (rural, urban), education level (primary, secondary, third), marital status (married, others), smoking status (current, former, never), current drinking (yes, no), BMI (continuous), hypertension (yes, no) and diabetes (yes, no) were further adjusted. Then, we evaluated the effect of the TyG index on CVD events stratified by hsCRP level, and vice versa.

Subgroup analyses were conducted by age groups: 45–59 years, 60–69 years, and ~ 70 years. Three sensitivity analyses were conducted as follows: (1) we used 1.0 mg/L and 3.0 mg/L as the cutoff points of hsCRP [35]; thus, participants were divided into six groups together with the TyG index; (2) all analyses were repeated using the complete data set (7126 participants) without multiple imputations; and (3) we created 5 imputed data sets and pooled the results using multiple imputation of the chained equation Markov chain Monte Carlo method.

We conducted a mediation analysis to assess the direct and indirect associations between the TyG index and CVD events via higher hsCRP. In brief, a higher TyG index (≥ median value of 8.6) was used as a predictor variable (X), a higher hsCRP level (≥ 1.0 mg/L) was used as a mediator (M) and a CVD event was used as the outcome variable (Y), as widely used in previous studies to quantify the mediating effect [36]. Meanwhile, the mediating effect of hsCPR on CVD events through the TyG index was similarly evaluated.

All statistical analyses were performed using R software (version 4.2.1). Multiple imputation was performed using the ‘mice’ package. Mediation analysis was performed using the ‘mediation’ package, and Cox regression analysis was performed using the ‘survival’ package. A two-sided P value < 0.05 was considered statistically significant.

## Results

A total of 8 658 participants in CHARLS from 2011 to 2018 were included in the final analyses. The mean (SD) age was 58.6 (9.0) years, including 3988 (46.1%) females. Table 1 and Additional file 1: Table S1 show the characteristics of the participants. At baseline, 1826 (21.1%) participants had a solely elevated TyG index, 1917 (22.1%) participants had a solely elevated hsCRP, and 2503 (28.9%) participants had both an elevated TyG index and hsCRP level. Compared to participants with low TyG and low hsCRP levels, those with high TyG index and hsCRP levels were more likely to be older, male, living in urban areas, and having diabetes. Additional file 1: Fig. S2 presents the codistribution of the TyG index and hsCRP stratified by incident CVD. Participants with CVD events were more likely to have both high TyG and hsCRP levels.

**Table 1:** Characteristics of 8 658 participants according to TyG and hsCRP levels

Characteristics	Overall	TyG < median & hsCRP < 1 mg/L	TyG ≥ median & hsCRP ≥ 1 mg/L	P value
Participants, No	8658	2412	2503	
Age, years, mean (SD)	58.58 (9.01)	57.78 (8.96)	59.18 (8.92)	< 0.001
Sex, Female, n (%)	3988 (46.1)	1165 (48.3)	1037 (41.4)	< 0.001
Residence, n (%)				< 0.001
Rural	6976 (80.7)	2023 (84.0)	1891 (75.6)	
Urban	1672 (19.3)	386 (16.0)	611 (24.4)	
Marriage, married, n (%)	7754 (89.6)	2170 (90.0)	2231 (89.1)	0.108
Educational level, n (%)				0.603
Primary	5833 (67.4)	1632 (67.7)	1668 (66.7)	
Secondary	1844 (21.3)	511 (21.2)	525 (21.0)	
Third	975 (11.3)	269 (11.2)	308 (12.3)	
Smoking status, n (%)				0.263
Never	5157 (59.6)	1451 (60.3)	1513 (60.5)	
Former	629 (7.3)	162 (6.7)	199 (8.0)	
Current	2862 (33.1)	794 (33.0)	789 (31.5)	
Current drinking, n (%)	2991 (34.6)	817 (34.0)	857 (34.3)	0.17
BMI^a^, kg/m^2^				
Continuous	23.76 (6.03)	23.89 (6.20)	23.85 (6.05)	0.333
< 23.9	4018 (56.2)	1129 (56.5)	1142 (55.4)	0.861
24–27.9	1559 (21.8)	419 (21.0)	467 (22.6)	
≥ 28	1567 (21.9)	452 (22.6)	453 (22.0)	
Hypertension, n (%)	3704 (42.8)	1045 (43.3)	1069 (42.7)	0.905
Antihypertensive, n (%)	1289 (14.9)	363 (15.0)	353 (14.1)	0.520
Diabetes, n (%)	1577 (18.2)	216 (9.0)	772 (30.8)	< 0.001
Antidiabetic, n (%)	254 (2.9)	71 (2.9)	77 (3.1)	0.953

Data are presented as the mean (SD) or number (%), as appropriate. Number of missing: BMI (n = 1514); residence (n = 10); smoking (n = 10); drinking (n = 13); education (n = 6). Median TyG index: 8.6

*SD* standard deviation, *BMI* body mass index, *TyG* triglyceride-glucose index, *hsCRP* high-sensitivity C-reactive protein

^a^Calculated as weight in kilograms divided by height in meters squared

**Fig. 1:**
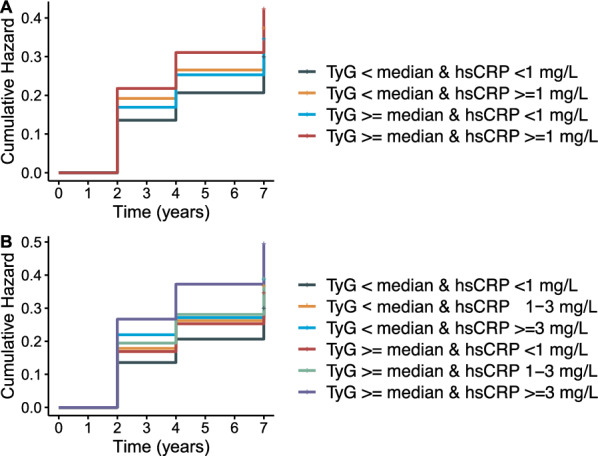
K‒M plot of cardiovascular diseases by TyG index and hsCRP level. *TyG* triglyceride-glucose index, *hsCRP* high-sensitivity C-reactive protein; median of TyG index: 8.6. **a** hsCRP was divided into two groups at 1 mg/L; **b** hsCRP was divided into three groups at 1 and 3 mg/L

During a maximum follow-up of 7.0 years, 2606 (30.1%) people developed cardiovascular diseases, including 2012 (23.2%) cases of heart diseases and 848 (9.8%) cases of stroke. Additional file 1: Table S2 shows the effects of individual exposures of the TyG index and hsCRP level on CVD onset. The incidence rates of CVD were 41.5 per 1000 years among participants with both low TyG and hsCRP levels, 52.2 per 1000 years among participants with low TyG but high hsCRP levels, 48.3 per 1000 years among participants with low hsCRP levels but high TyG, and 59.2 per 1000 years among participants with high TyG and hsCRP levels. Figure 1 displays the Kaplan–Meier curves of the cumulative incidence of CVD in the overall study participants. Additional file 1: Fig. S3 presents the Kaplan–Meier curves of the cumulative incidence of coronary heart diseases and stroke. Table 2 shows the associations of the TyG index and hsCRP with incident CVD events. After adjusting for potential confounders (in model 2), people with solely elevated hsCPR, solely elevated TyG, and elevated hsCPR plus TyG were independently associated with a 19.3% (adjusted HR [aHR], 1.193; 95% CI 1.053–1.351), 16.8% (aHR, 1.168; 95% CI 1.027–1.327), and 30.0% (aHR, 1.300; 95% CI 1.155–1.462) increased risk of incident CVD compared to those with low hsCRP and TyG index. Similar results were found for coronary heart disease, while only people with both higher TyG and hsCRP levels had a significantly increased risk of stroke. Additional file 1: Table S3 shows the effects of coexposures of the TyG index and hsCRP level on CVD onset when hsCRP was divided into three groups.

**Table 2:** Risk of cardiovascular disease upon coexposure stratified by the TyG index and hsCRP

	Model 1		Model 2	
	HR (95% CI)	P value	HR (95% CI)	P value
CVD (cases/person-years)				
Group 1 (620/14922)	Ref			
Group 2 (595/11408)	1.234 (1.102–1.381)	< 0.001	1.193 (1.053–1.351)	0.006
Group 3 (531/10990)	1.157 (1.03–1.299)	0.014	1.168 (1.027–1.327)	0.018
Group 4 (860/14537)	1.387 (1.25–1.538)	< 0.001	1.300 (1.155–1.462)	< 0.001
CHD (cases/person-years)				
Group 1 (463/14922)	Ref			
Group 2 (465/11408)	1.292 (1.136–1.47)	< 0.001	1.245 (1.079–1.436)	0.003
Group 3 (413/10990)	1.194 (1.045–1.363)	0.009	1.189 (1.026–1.377)	0.021
Group 4 (671/14537)	1.425 (1.266–1.605)	< 0.001	1.294 (1.130–1.481)	< 0.001
Stroke (cases/person-years)				
Group 1 (216/14922)	Ref			
Group 2 (189/11408)	1.090 (0.896–1.326)	0.388	1.115 (0.900–1.380)	0.318
Group 3 (165/10990)	1.047 (0.855–1.283)	0.656	1.067 (0.854–1.335)	0.568
Group 4 (278/14537)	1.298 (1.086–1.551)	0.004	1.333 (1.093–1.628)	0.005

*HR* hazard ratio, *CI* confidence interval, *BMI* body mass index, *TyG* triglyceride-glucose index, *hsCRP*: high-sensitivity C-reactive protein, *CVD* cardiovascular disease, *CHD* coronary heart disease

Group 1: TyG < median & hs-CRP < 1 mg/L; Group 2: TyG < median & hs-CRP ≥ 1 mg/L; Group 3: TyG ≥ median & hs-CRP < 1 mg/L; Group 4: TyG ≥ median & hs-CRP ≥ 1 mg/L. Median TyG index: 8.6

Model 1: age and sex were adjusted; model 2: age, sex, residence, marriage, education level, BMI level, smoking status, current drinking, hypertension and diabetes were adjusted

**Fig. 2:**
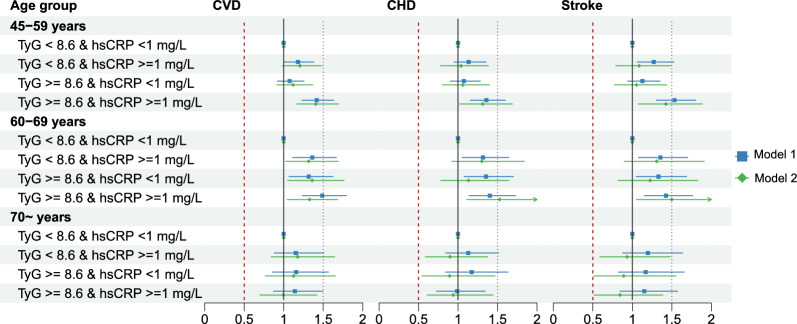
Age-associated risk of TyG index and hsCRP for cardiovascular disease onset. *HR* hazard ratio, *CI* confidence interval, *BMI* body mass index, *TyG* triglyceride-glucose index, *hsCRP* high-sensitivity C-reactive protein, *CVD* cardiovascular disease, *CHD* coronary heart disease. Number of participants: 45–59 years (n = 5 015); 60–69 years (n = 2 495); 70 ~ years (n = 1 148). Dots and lines represent the HR and 95% CI. Model 1: age and sex were adjusted; model 2: age, sex, residence, marriage, education level, BMI level, smoking status, current drinking, hypertension and diabetes were adjusted

Regardless of hsCRP level, participants with a higher TyG index had a significantly increased risk of CVD events. Similarly, participants with higher hsCRP levels had a significantly increased risk of CVD events independent of the TyG index, as shown in Table 3. In terms of age groups, the effect of coexposure of the TyG index and hsCRP tended to be predominant among those aged 70 years old or younger, as shown in Fig. 2 and Additional file 1: Fig. S4. The results remained consistent among the complete data set without missing data (7126 participants) and multiple imputed data sets (Additional file 1: Table S4). In addition, we repeated the analysis among those free of diabetes, and the associations between joint TyG and hsCRP groups with CVD events were consistent (Additional file 1: Table S5). After further adjusting for the uses of antihypertensive and antidiabetic medications, we found that the association magnitudes were slightly exaggerated compared to the main analysis (Additional file 1: Table S6).

**Table 3:** Risk reclassification of cardiovascular disease based on the TyG index and hsCRP

	CVD		CHD		Stroke	
	HR (95% CI)	P value	HR (95% CI)	P value	HR (95% CI)	P value
Scenario 1 (N of participants)						
hsCRP < 1 mg/L (N = 4 238)						
TyG < median	–		–		–	
TyG ≥ median	1.159 (1.032–1.301)	0.013	1.191 (1.043–1.361)	0.010	1.058 (0.863–1.296)	0.587
hsCRP ≥ 1 mg/L (N = 4 420)						
TyG < median	–				–	
TyG ≥ median	1.121(1.009–1.245)	0.034	1.105 (0.981–1.245)	0.101	1.171 (0.972–1.411)	0.097
Scenario 2 (N of participants)						
TyG < median (N = 4 329)						
hsCRP < 1 mg/L	–		–		–	
hsCRP ≥ 1 mg/L	1.229 (1.098–1.377)	< 0.001	1.285 (1.129–1.463)	< 0.001	1.082 (0.889–1.316)	0.434
TyG ≥ median (N = 4 329)						
hsCRP < 1 mg/L	–		–		–	
hsCRP ≥ 1 mg/L	1.198 (1.075–1.336)	0.001	1.196 (1.058–1.352)	0.004	1.243 (1.024–1.507)	0.027

*HR* hazard ratio, *CI* confidence interval, *BMI* body mass index, *TyG* triglyceride-glucose index, *hsCRP*: high-sensitivity C-reactive protein, *CVD* cardiovascular disease, *CHD* coronary heart disease. Median TyG index: 8.6

Model 1: age and sex were adjusted; model 2: age, sex, residence, marriage, education level, BMI level, smoking status, current drinking, hypertension and diabetes were adjusted

Scenario 1: effect of TyG on cardiovascular diseases between hsCRP groups; scenario 2: effect of hsCRP on cardiovascular diseases between TyG groups

**Fig. 3:**
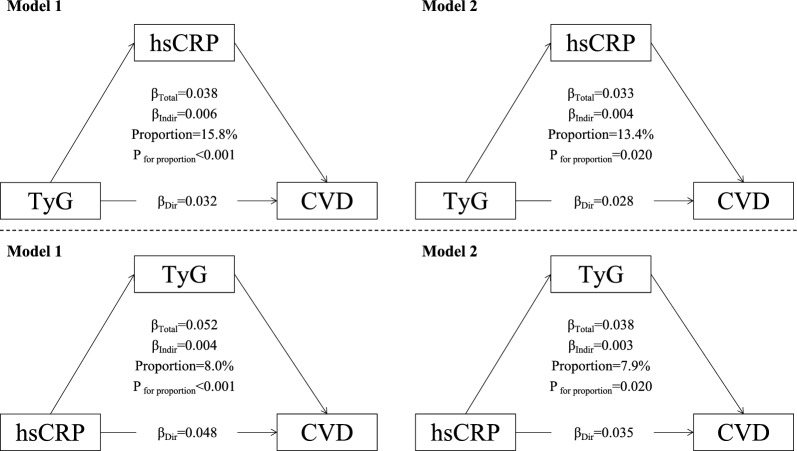
Mutual mediation effects of the TyG index and hsCRP on cardiovascular diseases. *TyG* triglyceride-glucose index, *hsCRP* high-sensitivity C-reactive protein. Model 1: age and sex were adjusted; model 2: age, sex, residence, marriage, education level, BMI level, smoking status, current drinking, hypertension and diabetes were adjusted

Figure 3 summarizes the mutual mediating effects linking TyG and hsCRP to CVD events. High hsCRP significantly mediated 13.4% (P = 0.020) of the association between a high TyG index and CVD events, while TyG simultaneously mediated 7.9% (P = 0.020) of the association between high hsCRP and cardiovascular risk in the fully adjusted model. In terms of coronary heart disease, the mediation proportions were 16.9% (P = 0.020) and 7.4% (P < 0.001), respectively (Additional file 1: Fig. S5). In terms of stroke, the mediation proportions were similar at 15.7% (P = 0.240) and 7.8% (P = 0.180), although the mutual mediation effects between TyG and hsCRP were not statistically significant (Additional file 1: Fig. S6). In terms of the predictive capacity of TyG index and hsCRP, we found that combining TyG and hsCRP could slightly improve the C-statistics than TyG or hsCRP alone, which suggests that TyG and hsCRP are mutually correlated (Additional file 1: Fig. S7).

## Discussion

Among 8658 Chinese adults aged 45 years or above followed up to 7.0 years, coexposure to a higher TyG index and hsCRP was significantly associated with the highest risk of CVD events, especially among those aged 70 years or below. The TyG index can further restratify cardiovascular risk independently of hsCRP level, and vice versa. The associations persisted even after adjustment for other established cardiovascular risk factors. Importantly, our findings indicated a mutual mediation relationship between the TyG index and hsCRP in terms of atherosclerotic risk.

The positive associations of individual TyG index or hsCPR level with cardiovascular risk have been widely evaluated in previous studies. The Tehran Lipid and Glucose Study, the Kailuan Study, the National Health Insurance Service Study in Korea, and the Atherosclerosis Risk in Communities (ARIC) Study have reported that elevated levels of the baseline or long-term TyG index are associated with an increased risk of CVD events [37–42]. The cumulative exposure, variability, and progressive trajectory of the TyG index have also been linked with a higher risk of CVD events [40, 43–45]. In addition, there is an association of the TyG index with the established risk factors for CVD in several cohort studies. The Hanzhong Adolescent Hypertension Cohort study and our previous study found that the TyG index was independently associated with elevated arterial stiffness, which is an important predictor of CVD events [17, 46]. In addition, the effects of TyG on diabetes, carotid atherosclerosis, hypertension, liver diseases, renal function and frailty have also been reported [47–52].

On the other hand, chronic inflammation is another important risk factor for CVD. The role of inflammation in the propagation of atherosclerosis and susceptibility to CVD events is well established [53]. Of the wide array of inflammatory markers, hsCRP has received the most attention for its use in screening and risk reclassification of CVD. The Emerging Risk Factor Collaboration (ERFC) reviewed the association among hsCRP levels, CV risk factors, and vascular risk in 160,309 individuals from 54 prospective studies and found that hsCRP concentration is significantly associated with the risk of coronary heart disease, ischemic stroke, and vascular mortality [54]. A hsCRP level > 3 mg/L was independently associated with a 60% excess risk of the onset of coronary heart disease compared with levels < 1 mg/L after adjustment for all Framingham risk variables [55]. In the middle-aged Chinese population, hsCRP was associated with an increased risk of developing CVD [56].

There is an elusive link between insulin resistance and inflammation. Inflammation plays a key role in diabetogenesis and enhancing insulin resistance [25]. Inflammation also greatly mediates lipid metabolism, modifying the constitution and fraction of lipid profiles and deteriorating glucose levels [57], which constitute the current form of the TyG index. A recent study pointed out that inflammation assessed by hsCRP was a stronger predictor for risk of future CVD events and death than cholesterol, suggesting that inflammation and hyperlipidemia jointly contribute to atherothrombotic disease and the combined use of aggressive lipid-lowering and inflammation-inhibiting therapies to further reduce atherosclerotic risk [20]. Moreover, hsCRP and the TyG index have been used to develop the CRP-TyG index (CTI), synchronously reflecting the inflammation and insulin resistance status given the close correlation between inflammation and insulin resistance [58]. There is also accumulating evidence indicating that chronic inflammation as measured by hsCRP is associated with insulin resistance and other features of insulin resistance syndrome and then leads to increased cardiovascular risk [59]. The joint assessment of inflammation and the TyG index has been evaluated in cancer prognosis [60], while the joint effect on cardiovascular risk needs more data. Zhang et al. reported that the cumulative value of TyG and hsCRP may better identify moderate-to-severe asymptomatic intracranial arterial stenosis, as well as its severity and numerical burden [61]. This current study highlighted the coexposure effect of the TyG index and hsCRP on CVD events. People concurrently with a higher TyG and hsCRP had the highest risk of overall cardiovascular disease, coronary heart disease and stroke, which were predominant among those aged 70 years or below. Similarly, Lan et al. highlighted the need for age-specific combined assessment and management of chronic inflammation and dyslipidemia in primary prevention against diabetes, particularly for young adults [62]. In addition, the assessment of hsCRP level further stratifies the risk of CVD events irrelevant of baseline TyG level, and vice versa. These findings highlighted the need for combined evaluation of the TyG index and hsCRP for the primary prevention of CVD events in clinical practice.

In terms of the mutual association, our results indicated a significant mediating effect of the TyG index on CVD events partially through inflammation biomarkers. Mirzababaei et al. reported that inflammation can play an important role in the relationship between body shape indices and cardiometabolic risk factors among overweight and obese women [63]. Li et al. reported that in diabetic chronic coronary syndrome patients, insulin resistance and systemic inflammation synergistically increased the risk of CVD events, and systemic inflammation partially mediated the association between insulin resistance and clinical outcomes [64]. In addition, our findings indicated that hsCRP is a risk factor for CVD events partially via higher TyG levels, which is consistent with a review summarizing that the proinflammatory phenotype contributes to cardiovascular insulin resistance [65]. Similar to the coexposure pattern widely recognized between inflammation and hyperlipidemia to atherothrombotic disease [20], the current study underlined the implications of joint contributions between inflammation and insulin resistance as determinants for CVD events.

There are possible mechanisms underlining the complex correlation among the TyG index or insulin resistance, inflammation, and cardiovascular damage. Generally, insulin resistance plays an important role in dyslipidemia, metabolic cardiac alterations, and myocardial and vascular stiffness damage [66]. The presence of hsCRP within most atherosclerotic plaques and all acute myocardial infarction lesions, coupled with binding of hsCRP to lipoproteins and its capacity for pro-inflammatory complement activation, suggests that hsCRP may contribute to the pathogenesis of CVD events [67]. Polarization of macrophages and lymphocytes toward a pro-inflammatory phenotype can contribute to the progression of insulin resistance, possibly through the renin–angiotensin–aldosterone system, sympathetic activation and incretin modulators (e.g., DPP-4) and immune responses [65, 68].

## Limitations

Several limitations of the current study should be acknowledged. First, owing to the observational study design, we could not confirm the causal association among the TyG index, inflammation biomarkers, and cardiovascular risk. However, both TyG and hsCRP have been validated widely as predictors of CVD events. The main aim of this study was to evaluate the coexposure effect and mutual mediation relationship between TyG and hsCRP in terms of cardiovascular risk. Second, we still cannot exclude the possibility of residual or unmeasured confounding bias, which could affect the estimation of effect size. Third, the diagnosis of cardiovascular events was self-reported. Medical records were not available in the CHARLS, and we were not able to differentiate the fatal and nonfatal CVD events. However, some other large-scale studies, such as the English Longitudinal Study of Aging, found that self-reported incident cardiovascular disease had good agreement with medical records. Fourth, we used the TyG index as a surrogate marker of insulin resistance. Further studies are needed for validation using data on measured insulin levels in other populations.

## Conclusions

In summary, using a prospective and national cohort of Chinese adults, we found that inflammation significantly mediated the association of insulin resistance with cardiovascular risk, and vice versa. The findings highlight the coexposure effect of the TyG index and hsCRP level in terms of cardiovascular events and recommend the combined assessment of the TyG index and inflammation markers to further stratify cardiovascular risk.

### Supplementary Information

**Additional file 1: Table S1.** Baseline characteristics of the study participants. **Table S2.** Risk of cardiovascular disease upon individual exposure stratified by TyG index and hsCRP. **Table S3.** Risk of cardiovascular disease upon co-exposure stratified by TyG index and hsCRP. **Table S4.** Sensitivity analyses of co-exposure for TyG index and hsCRP in terms of data missing. **Table S5.** Risk of cardiovascular disease upon coexposure stratified by the TyG index and hsCRP among those free of diabetes (7081 participants). **Table S6.** Risk of cardiovascular disease upon coexposure stratified by the TyG index and hsCRP further adjusting for medication uses. **Figure S1.** Flowchart and follow-up setting of this current study. **Figure S2.** Co-distribution of TyG index and hsCRP stratified by incident cardiovascular diseases or not. **Figure S3.** K-M plot of coronary heart disease and stroke by TyG index and hsCRP level. **Figure S4.** Age-associated risk of TyG index and hsCRP for cardiovascular diseases onset. **Figure S5.** Mutual mediation effects of TyG index and hsCRP on coronary heart disease. **Figure S6.** Mutual mediation effects of TyG index and hsCRP on stroke. **Figure S7.** Predictive capacity of TyG index and hsCRP on the cardiovascular risk.

## Data Availability

The data sets used and/or analyzed during the current study are publicly available or from the corresponding author upon reasonable request. All authors verify that all information and materials in the manuscript are original.

## Abbreviations

CHARLS: China Health and Retirement Longitudinal Study
TyG: Triglyceride-glucose index
hsCRP: High-sensitivity C-reactive protein
BMI: Body mass index
