# Supplementary material for: Joint association of TyG index and high sensitivity C-reactive protein with cardiovascular disease: a national cohort study
Source: Cardiovasc Diabetol. 2024 May 7;23:156. doi: 10.1186/s12933-024-02244-9 (PMC11077847; doi:10.1186/s12933-024-02244-9)
Supplement: Supplementary file 1 — Additional file 1: Table S1. Baseline characteristics of the study participants. Table S2. Risk of cardiovascular disease upon individual exposure stratified by TyG index and hsCRP. Table S3. Risk of cardiovascular disease upon co-exposure stratified by TyG index and hsCRP. Table S4. Sensitivity analyses of co-exposure for TyG index and hsCRP in terms of data missing. Table S5. Risk of cardiovascular disease upon coexposure stratified by the TyG index and hsCRP among those free of diabetes (7081 participants). Table S6. Risk of cardiovascular disease upon coexposure stratified by the TyG index and hsCRP further adjusting for medication uses. Figure S1. Flowchart and follow-up setting of this current study. Figure S2. Co-distribution of TyG index and hsCRP stratified by incident cardiovascular diseases or not. Figure S3. K-M plot of coronary heart disease and stroke by TyG index and hsCRP level. Figure S4. Age-associated risk of TyG index and hsCRP for cardiovascular diseases onset. Figure S5. Mutual mediation effects of TyG index and hsCRP on coronary heart disease. Figure S6. Mutual mediation effects of TyG index and hsCRP on stroke. Figure S7. Predictive capacity of TyG index and hsCRP on the cardiovascular risk. [file 12933_2024_2244_MOESM1_ESM.pdf]

Supplementary File

Joint association of TyG index and high sensitivity C-reactive protein with cardiovascular disease: a national cohort study

Table S1: Baseline characteristics of the study participants

Characteristics	Group 1	Group 2	Group 3	Group 4
Participants, No.	2412	1917	1826	2503
Age, years, mean (SD)	57.78(8.96)	59.58(9.38)	57.77(8.64)	59.18(8.92)
Sex, Female, n (%)	1165(48.3)	1016(53.0)	770(42.2)	1037(41.4)
Residence, n (%)				
Rural	2023(84.0)	1586(82.7)	1476(81.1)	1891(75.6)
Urban	386(16.0)	331(17.3)	344(18.9)	611(24.4)
Marriage, married, n (%)	2170(90.0)	1696(88.5)	1657(90.7)	2231(89.1)
Educational level, n (%)				
Primary	1632(67.7)	1300(67.8)	1233(67.6)	1668(66.7)
Secondary	511(21.2)	409(21.3)	399(21.9)	525(21.0)
Third	269(11.2)	207(10.8)	191(10.5)	308(12.3)
Smoking status, n (%)				
Never	1451(60.3)	1115(58.2)	1078(59.1)	1513(60.5)
Former	162(6.7)	134(7.0)	134(7.3)	199(8.0)
Current	794(33.0)	667(34.8)	612(33.6)	789(31.5)
Current drinking, n (%)	817(34.0)	703(36.7)	614(33.7)	857(34.3)
BMI [a], kg/m^2				
Continuous	23.89(6.20)	23.56(5.90)	23.67(5.91)	23.85(6.05)
<23.9	1129(56.5)	896(56.7)	851(56.6)	1142(55.4)
24-27.9	419(21.0)	341(21.6)	332(22.1)	467(22.6)
≥28	452(22.6)	342(21.7)	320(21.3)	453(22.0)
Hypertension, n (%)	1045(43.3)	809(42.2)	781(42.8)	1069(42.7)
Diabetes, n (%)	216(9.0)	170(8.9)	419(22.9)	772(30.8)

Data are presented as the mean (SD) or number (%), as appropriate.
Abbreviations: SD, standard deviation; BMI, body mass index; TyG, triglyceride-glucose index; hsCRP: high-sensitivity C-reactive protein.
[a] Calculated as weight in kilograms divided by height in meters squared.
N of missing: BMI (n=1514); residence (n=10); smoking (n=10); drinking (n=13); education (n=6).
Group 1: TyG < median & hs-CRP <1 mg/L; Group 2: TyG < median & hs-CRP ≥1 mg/L; Group 3: TyG ≥ median & hs-CRP <1 mg/L; Group 4: TyG ≥ median & hs-CRP ≥1 mg/L.

Table S2: Risk of cardiovascular disease upon individual exposure stratified by TyG index and hsCRP

	Model 1		Model 2	
	HR (95% CI)	P value	HR (95% CI)	P value
TyG < median	Ref			
TyG ≥ median				
CVD	1.17(1.083-1.264)	<0.001	1.146(1.049-1.252)	0.003
CHD	1.177(1.078-1.286)	<0.001	1.128(1.019-1.248)	0.020
Stroke	1.146(1.001-1.312)	0.049	1.159(0.995-1.35)	0.058
hsCRP <1 mg/L	Ref			
hsCRP ≥1 mg/L				
CVD	1.238(1.145-1.337)	<0.001	1.167(1.071-1.272)	<0.001
CHD	1.263(1.156-1.380)	<0.001	1.176(1.065-1.297)	0.001
Stroke	1.182(1.032-1.354)	0.016	1.199(1.034-1.391)	0.016

Abbreviations: HR, hazard ratio; CI, confidence interval; BMI, body mass index; TyG, triglyceride-glucose index; hsCRP: high-sensitivity C-reactive protein; CVD: cardiovascular disease; CHD: coronary heart disease.

Group 1: TyG < median & hs-CRP <1 mg/L; Group 2: TyG < median & hs-CRP ≥1 mg/L; Group 3: TyG ≥ median & hs-CRP <1 mg/L; Group 4: TyG ≥ median & hs-CRP ≥1 mg/L. Median of TyG: 8.6

Model 1: age and sex adjusted; model 2: age, sex, residence, marriage, education level, BMI level, smoking status, current drinking, hypertension, diabetes were adjusted.

Table S3: Risk of cardiovascular disease upon co-exposure stratified by TyG index and hsCRP

	Model 1		Model 2	
	HR (95% CI)	P value	HR (95% CI)	P value
CVD				
Group 1	Ref			
Group 2	1.210(1.066-1.374)	0.003	1.192(1.036-1.370)	0.014
Group 3	1.284(1.096-1.503)	0.002	1.196(1.001-1.430)	0.049
Group 4	1.157(1.030-1.299)	0.014	1.170(1.029-1.330)	0.016
Group 5	1.279(1.139-1.436)	<0.001	1.200(1.054-1.368)	0.006
Group 6	1.613(1.410-1.846)	<0.001	1.515(1.300-1.766)	<0.001
CHD				
Group 1	Ref			
Group 2	1.232(1.065-1.426)	0.005	1.189(1.012-1.397)	0.035
Group 3	1.415(1.186-1.688)	<0.001	1.362(1.118-1.659)	0.002
Group 4	1.194(1.045-1.363)	0.009	1.191(1.028-1.379)	0.020
Group 5	1.321(1.157-1.507)	<0.001	1.208(1.040-1.403)	0.013
Group 6	1.644(1.410-1.918)	<0.001	1.481(1.241-1.766)	<0.001
Stroke				
Group 1	Ref			
Group 2	1.130(0.909-1.405)	0.271	1.196(0.946-1.513)	0.135
Group 3	1.012(0.758-1.350)	0.936	0.955(0.690-1.321)	0.780
Group 4	1.047(0.855-1.283)	0.656	1.069(0.855-1.337)	0.559
Group 5	1.215(0.995-1.484)	0.056	1.239(0.993-1.546)	0.058
Group 6	1.468(1.160-1.858)	0.001	1.534(1.183-1.989)	0.001

Abbreviations: HR, hazard ratio; CI, confidence interval; BMI, body mass index; TyG, triglyceride-glucose index; hsCRP: high-sensitivity C-reactive protein; CVD: cardiovascular disease; CHD: coronary heart disease.

Group 1: TyG < median & hs-CRP <1 mg/L; Group 2: TyG < median & hs-CRP 1-3 mg/L; Group 3: TyG < median & hs-CRP ≥3 mg/L; Group 4: TyG ≥ median & hs-CRP ≥1 mg/L; Group 5: TyG ≥ median & hs-CRP 1-3 mg/L; Group 6: TyG ≥ median & hs-CRP ≥3 mg/L. Median of TyG: 8.6.

Model 1: age and sex adjusted; model 2: age, sex, residence, marriage, education level, BMI level, smoking status, current drinking, hypertension, diabetes were adjusted.

Table S4: Sensitivity analyses of co-exposure for TyG index and hsCRP in terms of data missing

	Sensitivity analysis 1		Sensitivity analysis 2	
	HR (95% CI)	P value	HR (95% CI)	P value
CVD				
Group 1	Ref			
Group 2	1.193(1.053-1.351)	0.006	1.229(1.098-1.376)	<0.001
Group 3	1.168(1.027-1.327)	0.018	1.138(1.012-1.279)	0.03
Group 4	1.300(1.155-1.462)	<0.001	1.341(1.205-1.491)	<0.001
CHD				
Group 1	Ref			
Group 2	1.245(1.079-1.436)	0.003	1.284(1.129-1.461)	<0.001
Group 3	1.189(1.026-1.377)	0.021	1.171(1.024-1.339)	0.021
Group 4	1.294(1.130-1.481)	<0.001	1.368(1.211-1.546)	<0.001
Stroke				
Group 1	Ref			
Group 2	1.115(0.901-1.380)	0.318	1.092(0.898-1.328)	0.378
Group 3	1.067(0.854-1.335)	0.568	1.027(0.837-1.260)	0.801
Group 4	1.333(1.093-1.628)	0.005	1.262(1.051-1.516)	0.013

Abbreviations: HR, hazard ratio; CI, confidence interval; BMI, body mass index; TyG, triglyceride-glucose index; hsCRP: high-sensitivity C-reactive protein; CVD: cardiovascular disease; CHD: coronary heart disease.

Group 1: TyG < median & hs-CRP <1 mg/L; Group 2: TyG < median & hs-CRP ≥1 mg/L; Group 3: TyG ≥ median & hs-CRP <1 mg/L; Group 4: TyG ≥ median & hs-CRP ≥1 mg/L. Median of TyG index: 8.6

Analyses were adjusted for age, sex, residence, marriage, education level, BMI level, smoking status, current drinking, hypertension, diabetes were adjusted.

Sensitivity analysis 1 was performed among 7 126 participants without any missing data; sensitivity analysis 2 were performed using multiple imputed analyses (5 iterations) by Markov chain Monte Carlo method.

Number of missing: BMI (n=1514); residence (n=10); smoking (n=10); drinking (n=13); education (n=6).

Table S5: Risk of cardiovascular disease upon coexposure stratified by the TyG index and hsCRP among those free of diabetes (7081 participants)

	Model 1		Model 2	
	HR (95% CI)	P value	HR (95% CI)	P value
CVD				
Group 1	Ref			
Group 2	1.255(1.115-1.413)	<0.001	1.227(1.077-1.398)	0.002
Group 3	1.15(1.012-1.308)	0.033	1.174(1.02-1.352)	0.026
Group 4	1.341(1.193-1.507)	<0.001	1.303(1.144-1.483)	<0.001
CHD				
Group 1	Ref			
Group 2	1.297(1.132-1.487)	<0.001	1.261(1.085-1.465)	0.003
Group 3	1.215(1.05-1.406)	0.009	1.229(1.047-1.444)	0.012
Group 4	1.389(1.215-1.588)	<0.001	1.305(1.124-1.516)	<0.001
Stroke				
Group 1	Ref			
Group 2	1.103(0.898-1.354)	0.35	1.132(0.906-1.416)	0.275
Group 3	0.942(0.748-1.187)	0.615	0.988(0.768-1.271)	0.927
Group 4	1.221(0.997-1.495)	0.053	1.323(1.062-1.648)	0.013

Abbreviations: HR, hazard ratio; CI, confidence interval; BMI, body mass index; TyG, triglyceride-glucose index; hsCRP: high-sensitivity C-reactive protein; CVD: cardiovascular disease; CHD: coronary heart disease.

Group 1: TyG < median & hs-CRP <1 mg/L; Group 2: TyG < median & hs-CRP ≥1 mg/L; Group 3: TyG ≥ median & hs-CRP <1 mg/L; Group 4: TyG ≥ median & hs-CRP ≥1 mg/L. Median of TyG index: 8.6

Analyses were adjusted for age, sex, residence, marriage, education level, BMI level, smoking status, current drinking, hypertension were adjusted.

Table S6: Risk of cardiovascular disease upon coexposure stratified by the TyG index and hsCRP further adjusting for medication uses

	CVD		CHD		Stroke	
	HR (95% CI)	P	HR (95% CI)	P	HR (95% CI)	P
Group 1	Ref					
Group 2	1.191(1.051-1.349)	0.006	1.243(1.078-1.434)	0.003	1.11(0.896-1.374)	0.338
Group 3	1.183(1.042-1.344)	0.009	1.207(1.043-1.397)	0.011	1.083(0.867-1.351)	0.483
Group 4	1.332(1.187-1.494)	0.001	1.327(1.163-1.515)	0.001	1.375(1.132-1.67)	0.001

Abbreviations: HR, hazard ratio; CI, confidence interval; BMI, body mass index; TyG, triglyceride-glucose index; hsCRP: high-sensitivity C-reactive protein; CVD: cardiovascular disease; CHD: coronary heart disease.

Group 1: TyG < median & hs-CRP <1 mg/L; Group 2: TyG < median & hs-CRP ≥1 mg/L; Group 3: TyG ≥ median & hs-CRP <1 mg/L; Group 4: TyG ≥ median & hs-CRP ≥1 mg/L. Median of TyG index: 8.6

Analyses were adjusted for age, sex, residence, marriage, education level, BMI level, smoking status, current drinking, hypertension, antihypertensive medication, diabetes and antidiabetic medication were adjusted.

Figure S1: Flowchart and follow-up setting of this current study

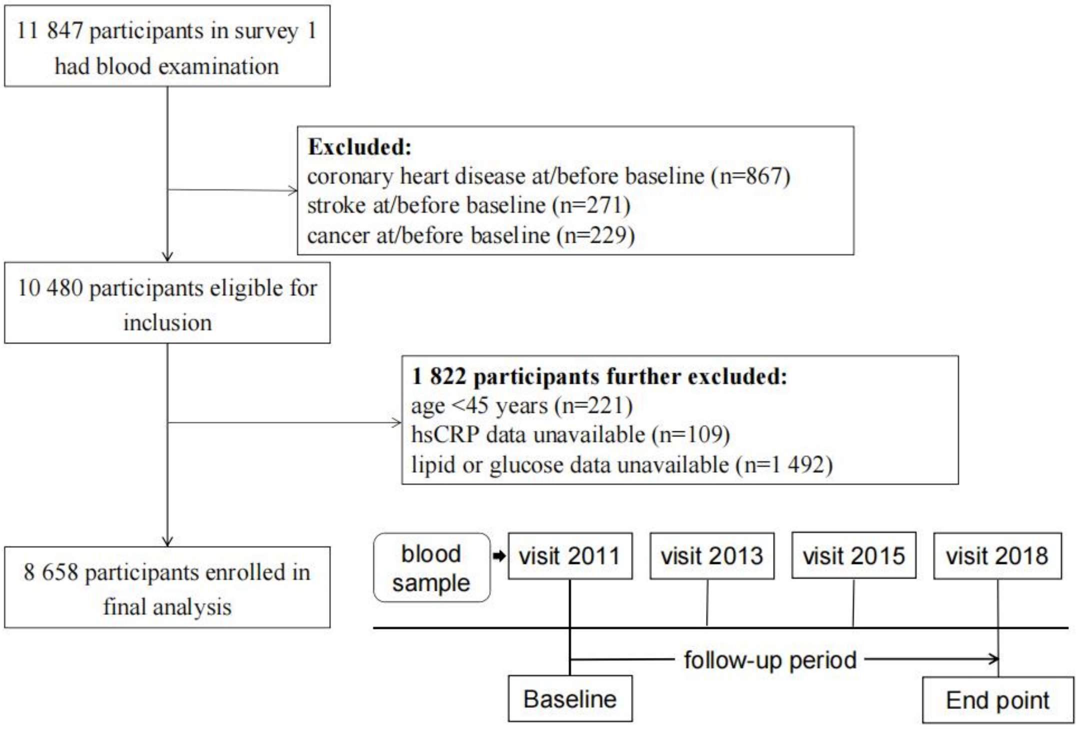

Figure S2: Co-distribution of TyG index and hsCRP stratified by incident cardiovascular diseases or not

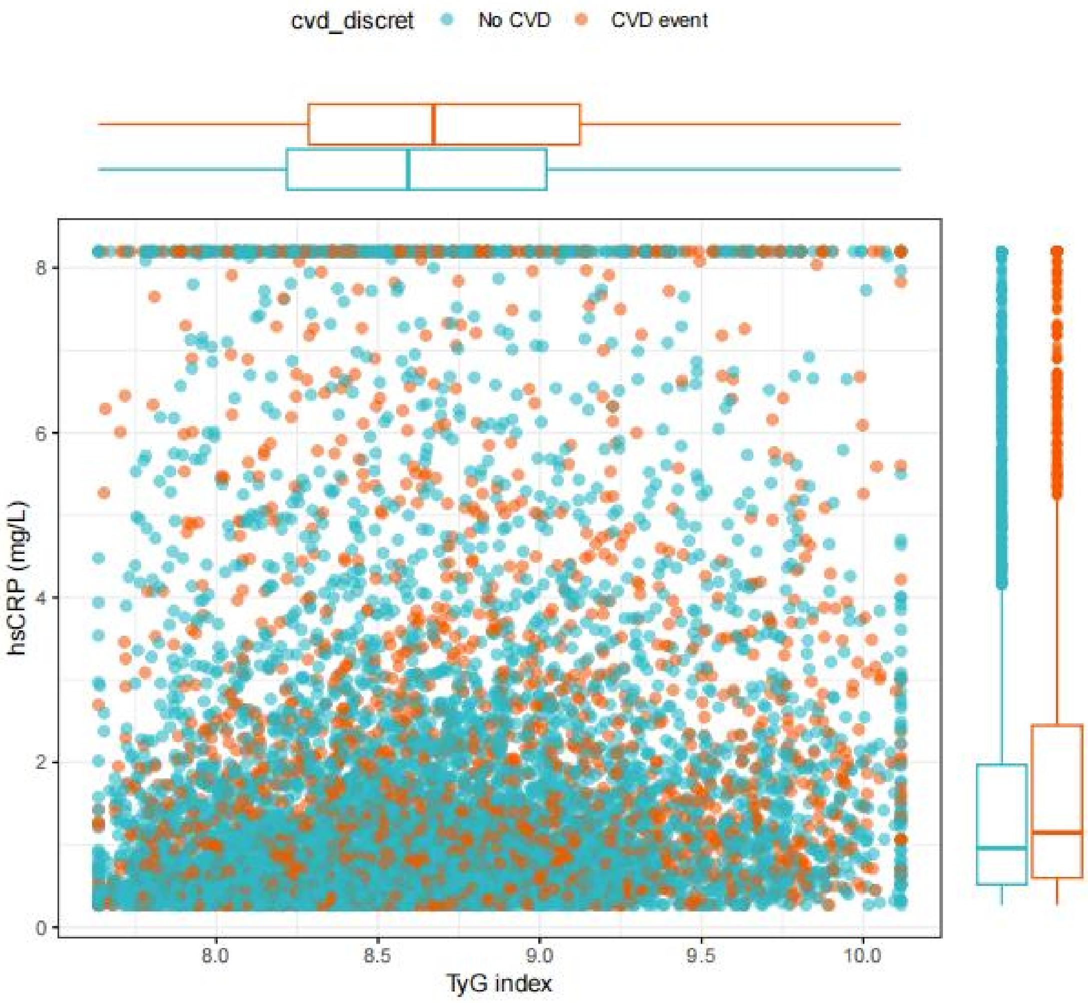

Abbreviations: TyG, triglyceride-glucose index; hsCRP: high-sensitivity C-reactive protein; CVD: cardiovascular disease

Figure S3: K-M plot of coronary heart disease and stroke by TyG index and hsCRP level

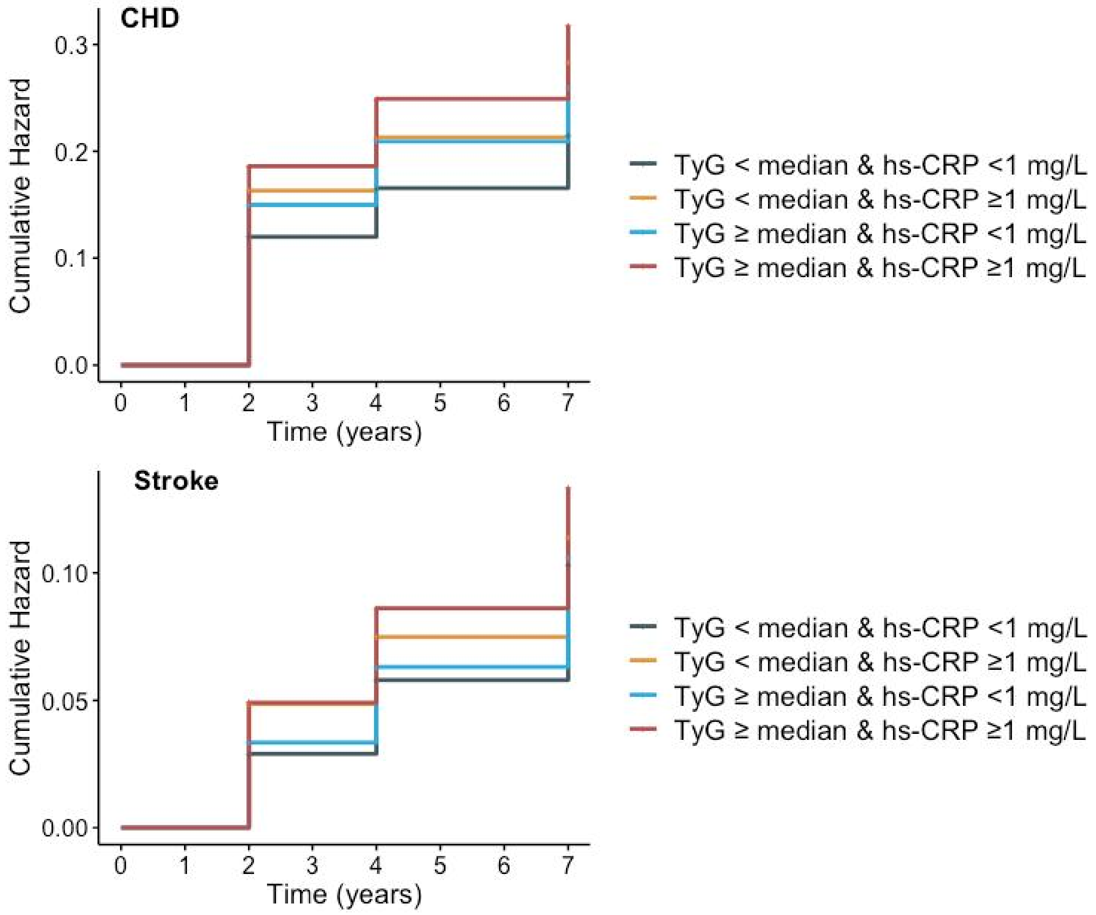

Abbreviations: TyG, triglyceride-glucose index; hsCRP: high-sensitivity C-reactive protein; median of TyG index: 8.6

Figure S4: Age-associated risk of TyG index and hsCRP for cardiovascular diseases onset

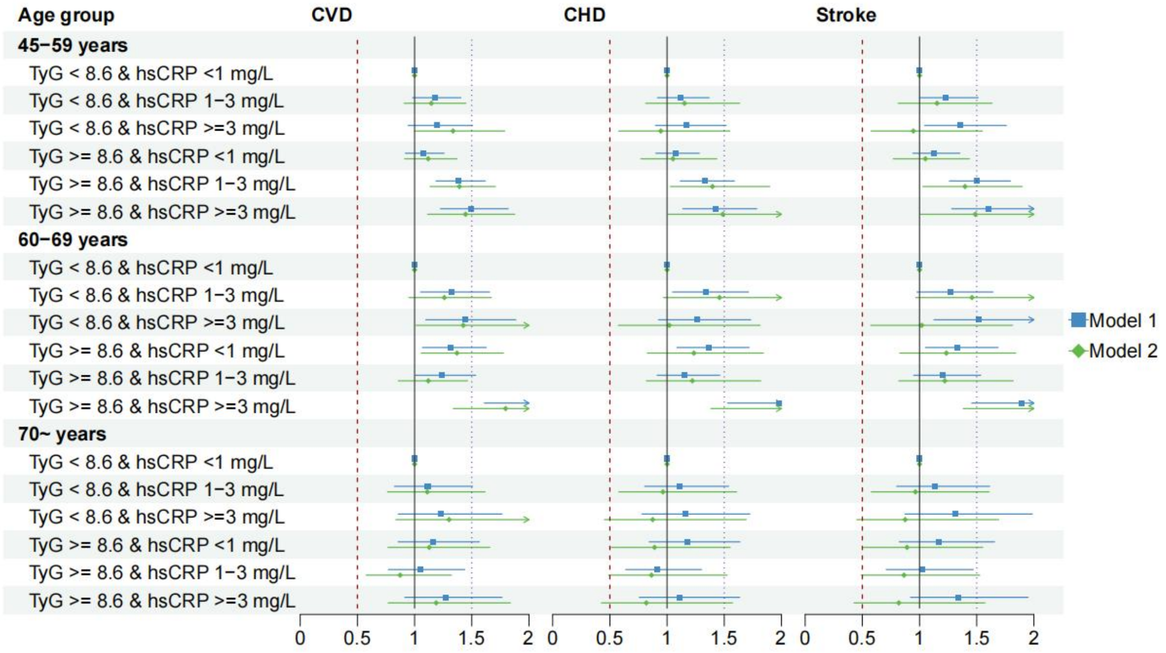

Abbreviations: HR, hazard ratio; CI, confidence interval; BMI, body mass index; TyG, triglyceride-glucose index; hsCRP: high-sensitivity C-reactive protein; CVD: cardiovascular disease; CHD: coronary heart disease.

Number of participants: 45-59 years (n=5 015); 60-69 years (n=2 495); 70~ years (n=1 148)

Dots and lines represent the HR and 95% CI.

Model 1: age and sex adjusted; model 2: age, sex, residence, marriage, education level, BMI level, smoking status, current drinking, hypertension, diabetes were adjusted.

Figure S5: Mutual mediation effects of TyG index and hsCRP on coronary heart disease

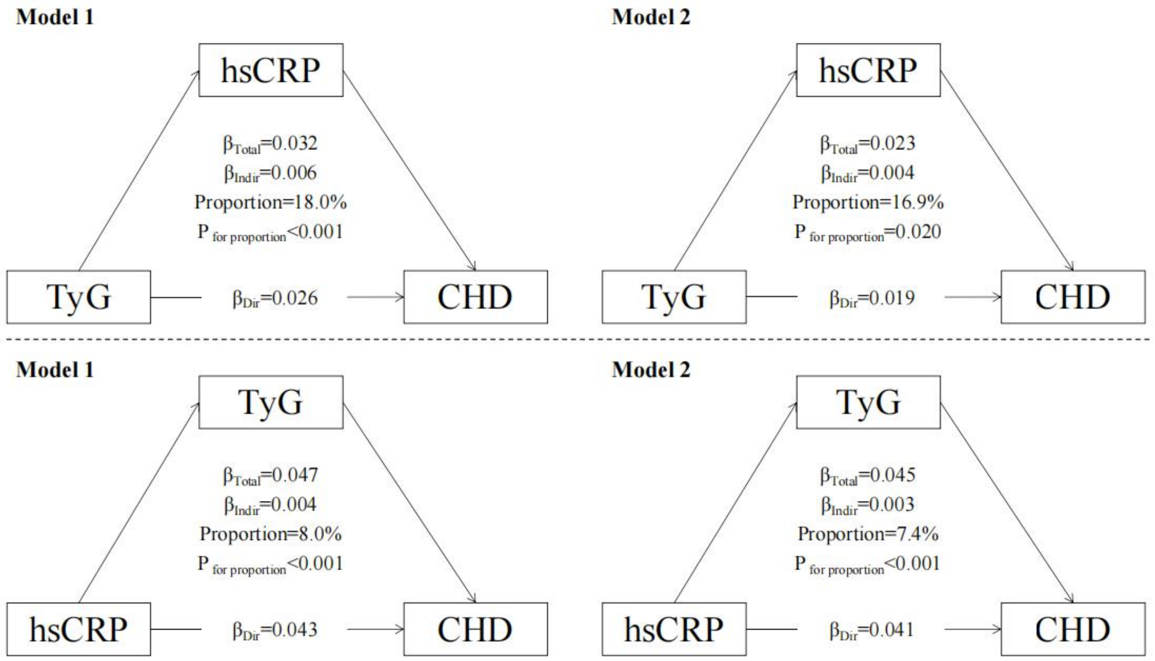

Abbreviations: TyG, triglyceride-glucose index; hsCRP: high-sensitivity C-reactive protein

Model 1: age and sex adjusted; model 2: age, sex, residence, marriage, education level, BMI level, smoking status, current drinking, hypertension, diabetes were adjusted.

Figure S6: Mutual mediation effects of TyG index and hsCRP on stroke

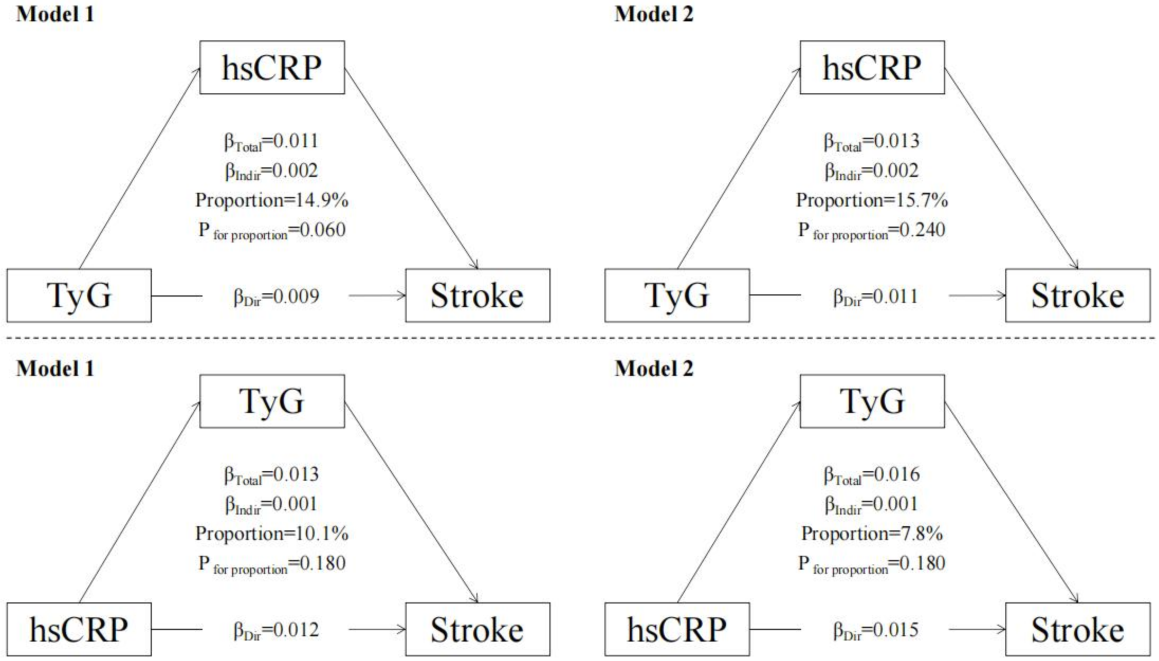

Abbreviations: TyG, triglyceride-glucose index; hsCRP: high-sensitivity C-reactive protein

Model 1: age and sex adjusted; model 2: age, sex, residence, marriage, education level, BMI level, smoking status, current drinking, hypertension, diabetes were adjusted.

Figure S7: Predictive capacity of TyG index and hsCRP on the cardiovascular risk

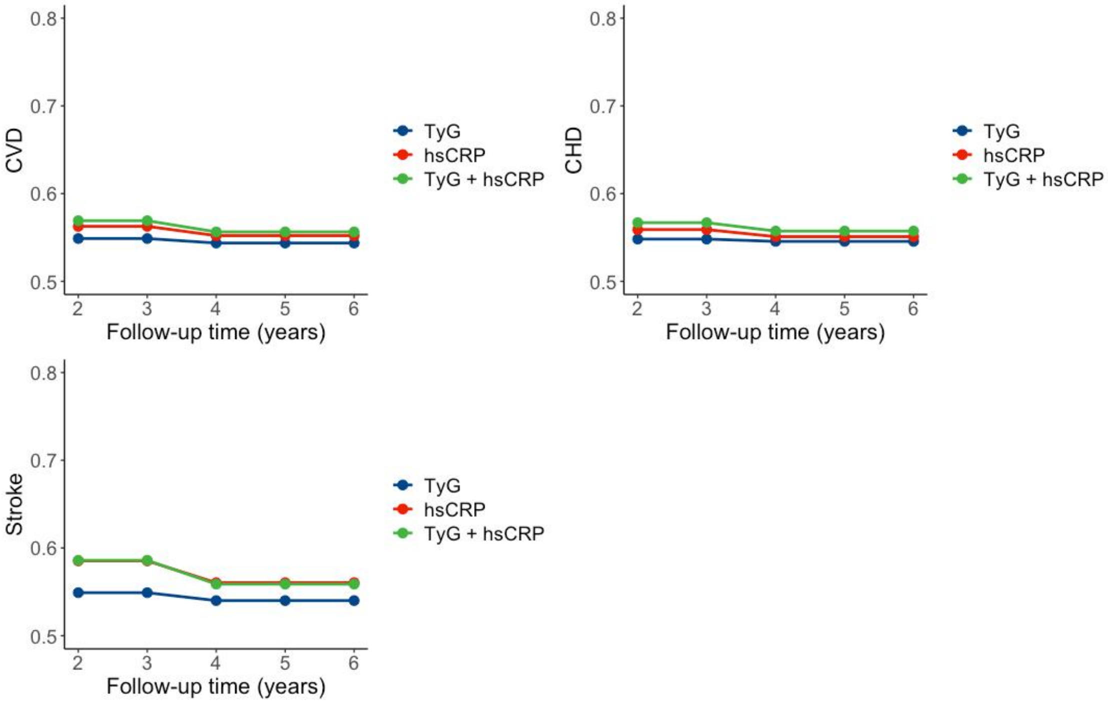

Abbreviations: TyG, triglyceride-glucose index; hsCRP: high-sensitivity C-reactive protein
